# Supplementary material for: Deubiquitinating Enzyme USP8 Is Essential for Skeletogenesis by Regulating Wnt Signaling
Source: Int J Mol Sci. 2021 Sep 24;22(19):10289. doi: 10.3390/ijms221910289 (PMC8508692; doi:10.3390/ijms221910289)
Supplement: Supplementary file 1 [file ijms-22-10289-s001.zip › ijms-1337401-supplementary.pdf]

Supplementary Materials

Deubiquitinating Enzyme USP8 is Essential for Skeletogenesis by Regulating Wnt Signaling

Sachin Chaugule [1], Jung-Min Kim [1], Yeon-Suk Yang [1], Klaus-Peter Knobeloch [2], Xi He [3] and Jae-Hyuck Shim [1,4,5,] *

1 Shim lab, Division of Rheumatology, Department of Medicine, University of Massachusetts Chan Medical School, Worcester, MA 01605, USA; sachin.chaugule@umassmed.edu (S.C.); jungmin.kim@umassmed.edu (J.-M.K.); yen.yang@umassmed.edu (Y.-S.Y.)

2 Institute of Neuropathology, Medical Faculty, University of Freiburg, 79106 Freiburg, Germany; klaus-peter.knobeloch@uniklinik-freiburg.de

3 F. M. Kirby Neurobiology Center, Boston Children's Hospital, Department of Neurology, Harvard Medical School, Boston, MA 02115, USA; xi.he@childrens.harvard.edu

4 Horae Gene Therapy Center, University of Massachusetts Chan Medical School, Worcester, MA 01605, USA

5 Li Weibo Institute for Rare Diseases Research, University of Massachusetts Chan Medical School, Worcester, MA 01605, USA

Supplementary Figure S1: *Usp8^Osx* mice display impaired ossification during skeletogenesis

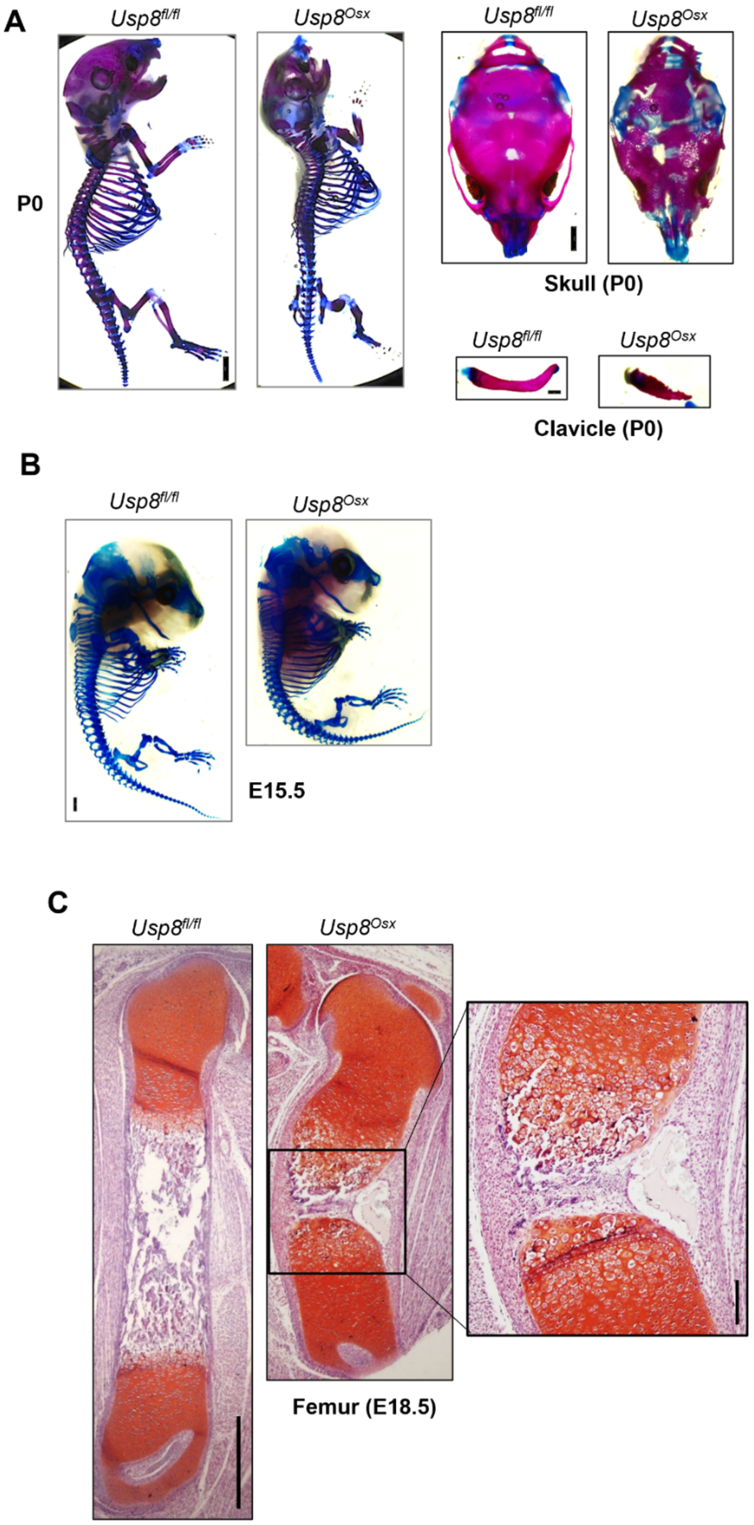

(A) Alizarin red/alcian blue staining of skeletal preparations of the whole body, skulls, and clavicles of P0 *Usp8^fl/fl* and *Usp8^Osx* embryos. Scale bar, 1 mm. (B) Alizarin red/alcian blue staining of skeletal preparations of the whole body of E15.5 *Usp8^fl/fl* and *Usp8^Osx* embryos. Scale bar, 1 mm. (C) Safranin O-stained longitudinal sections of E18.5 *Usp8^fl/fl* and *Usp8^Osx* femurs. Scale bar, 250 µm (left) and 100 µm (right, enlarged inset image).

Supplementary Figure S2: Isolation of mouse SSCs using flow cytometry

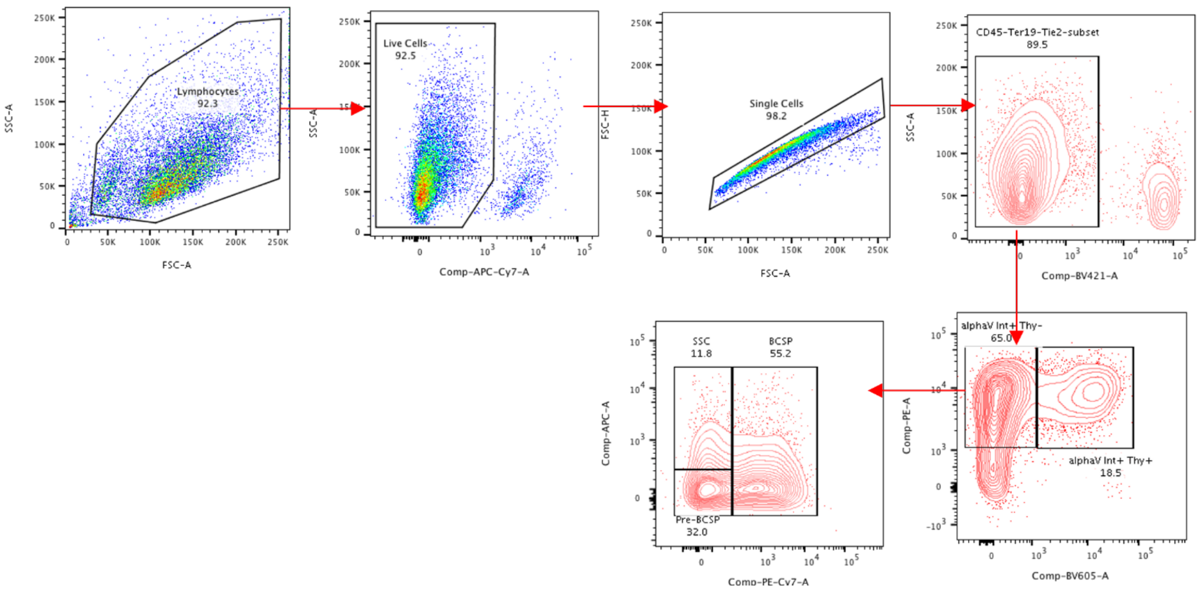

(A) Representative flow cytometry dot plots showing the gating strategy to isolate mouse skeletal stem cells (SSCs) from E18.5 *Usp8*$^{fl/fl}$ embryonic limbs. Dot plots showing the different populations while applying the gating strategy, such as BCSP (bone, cartilage, and stromal progenitor, CD45^{-}Ter119^{-}Tie2^{-}αVInt^{+}Thy^{+}CD105^{+}); pre-BCSP (pre-bone cartilage and stromal progenitor, CD45^{-}Ter119^{-}Tie2^{-}αVInt^{+}Thy^{-}CD105^{-}CD200^{-}) and SSC (skeletal stem cell, CD45^{-}Ter119^{-}Tie2^{-}αVInt^{+}Thy^{-}CD105^{-}CD200^{+}). A similar strategy was used to isolate SSCs from E18.5 *Usp8*^{Osx} embryonic limbs.

Supplementary Figure S3: The impaired osteogenic potential of *Usp8^{Osx}* calvarial osteoblasts.

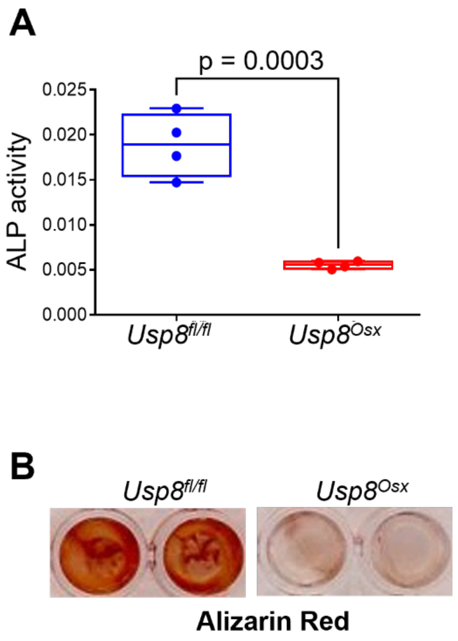

(A, B) Primary calvarial osteoblasts (COBs) isolated from *Usp8*$^{fl/fl}$ and *Usp8*^{Osx} neonates at postnatal day 0 were cultured under osteogenic conditions for 6 days, and ALP activity (A) and alizarin red staining (B) were performed to assess early and late osteoblast differentiation, respectively. Data are shown as a box-and-whisker plot (with median and interquartile ranges) from min to max, with all data points shown. Analysis was performed using the unpaired Student's t-test within Prism.

Supplementary Table S1: Number of offspring derived from the breeding of *Usp8*$^{fl/lf}$ and *Usp8*$^{fl/+}$*;Osx-cre* mice

Age	*Usp8*$^{fl/fl}$	*Usp8*$^{fl/+}$*;Osx*	*Usp8*$^{fl/fl}$*;Osx*	Total
P0	70	23	23	116
P21	124	46	0	170

Supplementary Table S2: Primer Sequences

Gene	Forward	Reverse
Mouse *Usp8*	CGCAATCATCTCCTTCCATT	GATTTGGGAGAAGTAGCCCC
Mouse *Col1a1*	ACTGTCCCAACCCCCAAAG	ACGTATTCTTCCGGGCAGAA
Mouse *Opn*	TGCACCCAGATCCTATAGCC	CTCCATCGTCATCATCATCG
Mouse *Osx*	ATGGCGTCCTCTCTGCTTGA	GAAGGGTGGGTAGTCATTTG
Mouse *Runx2*	TACAAACCATACCCAGTCCCTGTTT	AGTGCTCTAACCACAGTCCATGCA
